# Heat treatment of thioredoxin fusions increases the purity of α‐helical transmembrane protein constructs

**DOI:** 10.1002/pro.4150

**Published:** 2021-07-06

**Authors:** Mathias Schenkel, Antoine Treff, Charles M. Deber, Georg Krainer, Michael Schlierf

**Affiliations:** ^1^ B CUBE – Center for Molecular Bioengineering TU Dresden Dresden Germany; ^2^ Division of Molecular Medicine, Research Institute Hospital for Sick Children Toronto Ontario Canada; ^3^ Centre for Misfolding Diseases, Yusuf Hamied Department of Chemistry University of Cambridge Cambridge UK

**Keywords:** heat treatment, membrane proteins, protein purification, thioredoxin

## Abstract

Membrane proteins play key roles in cellular signaling and transport, represent the majority of drug targets, and are implicated in many diseases. Their relevance renders them important subjects for structural, biophysical, and functional investigations. However, obtaining membrane proteins in high purities is often challenging with conventional purification steps alone. To address this issue, we present here an approach to increase the purity of α‐helical transmembrane proteins. Our approach exploits the Thioredoxin (Trx) tag system, which is able to confer some of its favorable properties, such as high solubility and thermostability, to its fusion partners. Using Trx fusions of transmembrane helical hairpin constructs derived from the human cystic fibrosis transmembrane conductance regulator (CFTR) and a bacterial ATP synthase, we establish conditions for the successful implementation of the selective heat treatment procedure to increase sample purity. We further examine systematically its efficacy with respect to different incubation times and temperatures using quantitative gel electrophoresis. We find that minute‐timescale heat treatment of Trx‐tagged fusion constructs with temperatures ranging from 50 to 90°C increases the purity of the membrane protein samples from ~60 to 98% even after affinity purification. We show that this single‐step approach is even applicable in cases where regular selective heat purification from crude extracts, as reported for Trx fusions to soluble proteins, fails. Overall, our approach is easy to integrate into existing purification strategies and provides a facile route for increasing the purity of membrane protein constructs after purification by standard chromatography approaches.

## 1 INTRODUCTION

Membrane proteins are important actors in cross‐membrane cell physiology and fulfill a variety of functions ranging from cellular signaling to energy generation and transport.^1^, ^2^ They cover about 20–50% of prokaryotic and eukaryotic genomes,^2^ and constitute more than 50% of all approved drug targets.^3^ Many membrane proteins are involved in the pathogenesis of several severe diseases (e.g., cystic fibrosis) due to their misfolding from sequence mutations.^4^ Their abundance and therapeutic relevance, therefore, render them important subjects for structural, functional, and biophysical investigations in proteomics, interactomics, and in vitro drug screening workflows.^5^, ^6^

High sample purity is vital for studying membrane proteins and their respective segmental model constructs, such as single transmembrane helices and helical hairpins that preserve many characteristics of their full‐length counterparts.^7^, ^8^ In fact, in vitro investigations of membrane proteins, such as structural studies and single‐molecule experiments, typically demand milligram amounts of highly pure protein samples. For instance, X‐ray crystallography studies require sample purities of more than 90–95%.^9^ To achieve this, the production of membrane proteins typically involves overexpression of constructs in heterologous hosts^10^, ^11^ and purification by chromatography techniques such as, for example, immobilized metal affinity chromatography (IMAC).^12^ Prior to chromatographic separation, the protein of interest is usually extracted from the expression host cell membrane. This is achieved with adequate solubilization agents, such as detergents, to keep the hydrophobic proteins in solution as water‐soluble protein–detergent complexes in order to prevent aggregation.^7^, ^13^

To increase the solubility of membrane protein constructs, tags are often added to the protein of interest,^14^ among them thioredoxin (Trx),^15^, ^16^ a thermostable protein with a high melting temperature (*T*_m_) of ~85°C.^17^, ^18^ It was first presented by LaVallie et al.^19^ as a solubility fusion tag for cytokines and mammalian growth factors. Protocols have been established describing the construction of Trx gene fusions for expression as well as facile steps for purification of the Trx fusion proteins.^20^, ^21^ In addition to its role in increasing protein solubility, Trx‐tagged constructs can be purified by heat treatment due to their retained thermal stability from whole‐cell lysate.^19^ In fact, Trx can resist long incubations at high temperatures (80–90°C) without denaturing, a characteristic that is often preserved in Trx fusions and which enables heat treatments to be exploited as an effective purification method for Trx‐tagged proteins.^19^, ^20^, ^21^

The ability of Trx fusions to withstand heat treatment provides an opportunity to use this system as a tool for the purification of membrane proteins. Indeed, we have used the Trx tag to increase the purity of transmembrane–Trx fusion proteins in elution fractions gained after affinity chromatography. A helical hairpin peptide derived from the third and fourth transmembrane helix (TM3/4) of the human cystic fibrosis transmembrane conductance regulator (CFTR)^22^, ^23^ solubilized in detergent was subjected to heat treatment at 70°C for 10 min and subsequent centrifugation to obtain highly pure samples of Trx–TM3/4 fusion proteins. This exemplifies the simplicity and effectiveness of the Trx‐based selective heat treatment procedure for increasing the purity of helical membrane protein constructs. Yet a systematic evaluation and demonstration of the approach has been lacking.

Here we assess the efficiency of the heat treatment approach for the selective purification of Trx‐tagged transmembrane protein constructs and systematically examine its efficacy with respect to different incubation times and temperatures. As model systems, we evaluate Trx fusions of the CFTR‐derived mutant helical hairpin Q220R TM3/4,^24^ denoted as Trx–TM3/4^Q220R^, and the subunit C transmembrane peptide derived from the ATP synthase of *Ilyobacter tartaricus (I. tartaricus)*,^25^ denoted as Trx–subunit C. We use quantitative sodium dodecyl sulfate polyacrylamide gel electrophoresis (SDS‐PAGE) to determine optimal conditions for the heat treatment purification procedure and evaluate the impact of the heat treatment on secondary structure by circular dichroism (CD). We further probe if the fusion proteins can be selectively purified from whole‐cell crude extract, similar to previous protocols.^19^, ^20^, ^21^ We find that heat treatment increases the purity of affinity‐purified Trx transmembrane fusion constructs in elution fractions while retaining their native conformation. In conclusion, we establish conditions and guiding principles for the purification of Trx transmembrane fusion proteins by the heat treatment procedure. Our approach is easy to integrate into existing purification strategies and thus provides a facile route for obtaining purer samples for structural, biophysical, and biochemical studies on membrane protein constructs.

## 2 RESULTS AND DISCUSSION

**FIGURE 1:**
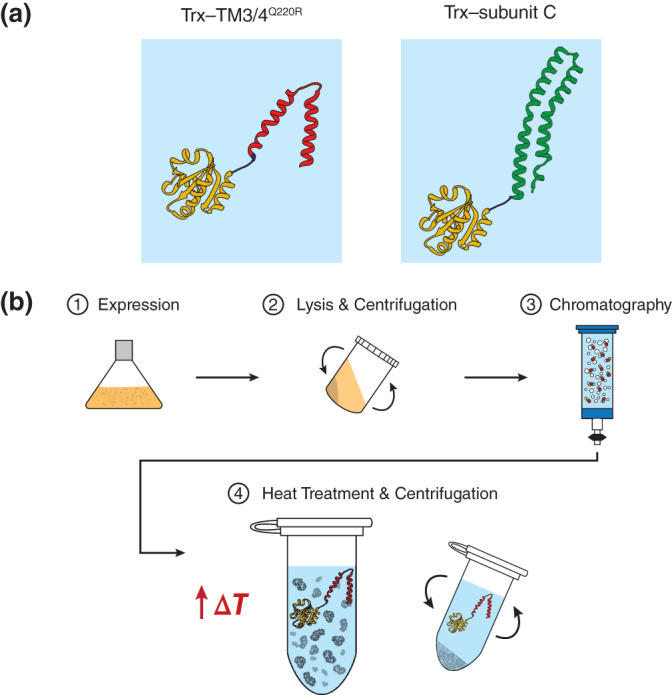
Heat treatment of Trx‐tagged α‐helical transmembrane constructs. (a) Representation of Trx‐tagged fusions (yellow, PDB ID: 2TRX)^30^ of the TM3/4^Q220R^ hairpin derived from human CFTR (red, PDB ID: 5UAK)^31^ (left panel) and the ATP synthase subunit C of *Ilyobacter tartaricus* (green, PDB ID: 1YCE)^32^ (right panel). Structures were created using UCSF Chimera (version 1.13.1).^36^ (b) Schematic of the heat treatment assay. *Step 1: Expression*. Trx fusion proteins are expressed heterologously in a suitable host (e.g., *E. coli*). *Step 2: Lysis and centrifugation*. Cells containing the fusion construct are lysed, followed by centrifugation to clarify the lysate. *Step 3*: *Chromatography*. Chromatography methods, such as affinity chromatography (e.g., IMAC), serve as an initial purification step to enrich the target protein and separate it from undesired and contaminating cellular proteins. *Step 4*: *Heat treatment and centrifugation*. Incubation at elevated temperatures and subsequent centrifugation yields high purity of Trx fusion proteins in the supernatant. Trx–TM3/4^Q220R^ is shown as an example for thermostable Trx fusion constructs. Protein contaminants are displayed in gray

**FIGURE 2:**
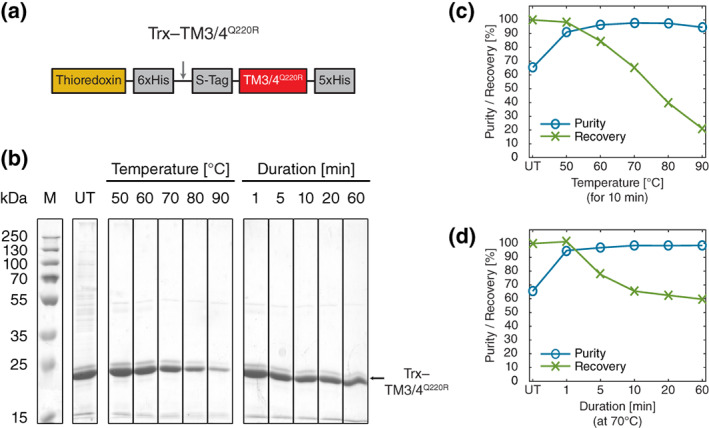
Heat treatment of the Trx–TM3/4^Q220R^ fusion construct. (a) Design of the Trx–TM3/4^Q220R^ construct. Trx is N‐terminally fused to the CFTR TM3/4 hairpin mutant via an internal His_6_‐Tag, a cleavage site for thrombin (gray arrow), and an S‐Tag. TM3/4^Q220R^ bears an additional C‐terminal His_5_‐tag. (b) 12% SDS‐PA gel with heat treatment series of a concentrated Trx–TM3/4^Q220R^ elution fraction after IMAC. Lanes show the untreated fraction (UT), heat incubations for 10 min at different temperatures, and different incubation durations at 70°C. Molecular weight marker (M) and expected molecular weight of the Trx–TM3/4^Q220R^ band (~23.5 kDa, black arrow) are indicated. (c) Purity and recovery of Trx–TM3/4^Q220R^ after heat treatment at different temperatures for 10 min. Values are normalized with respect to the untreated sample. (d) Purity and recovery of Trx–TM3/4^Q220R^ after heat treatment for different incubation durations at 70°C. Values are normalized with respect to the untreated sample

**FIGURE 3:**
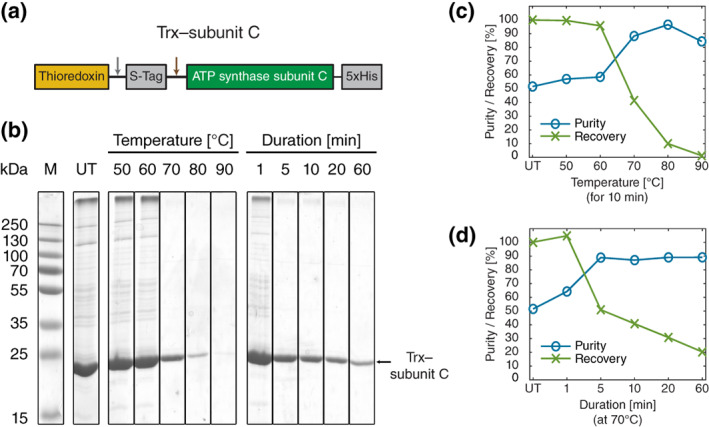
Heat treatment of the Trx–subunit C fusion construct. (a) Design of the Trx–subunit C construct. Trx is N‐terminally fused to the ATP synthase subunit C of *Ilyobacter tartaricus* via an internal S‐Tag and cleavage sites for thrombin and enterokinase (cleavage positions are indicated with gray and brown arrows, respectively). Subunit c bears an additional C‐terminal His_5_‐tag. (b) 12% SDS‐PA gel with heat treatment series of a concentrated Trx–subunit C elution fraction after IMAC. Lanes show the untreated fraction (UT), heat incubations for 10 min at different temperatures, and different incubation durations at 70°C. Molecular weight marker (M) and expected molecular weight of the Trx‐subunit C band (~25.8 kDa, black arrow) are indicated. (c) Purity and recovery of Trx–subunit C after heat treatment at different temperatures for 10 min. Values are normalized to the untreated sample. (d) Purity and recovery of Trx–subunit C after heat treatment for different incubation durations at 70°C. Values are normalized with respect to the untreated sample

To examine the efficiency of selective heat treatment and determine optimal conditions for the purification procedure, we evaluated the heat stability of purified Trx–TM3/4^Q220R^ and Trx–subunit C fusion proteins (Figure 1a) at different temperatures and incubation times. A schematic of the heat treatment procedure is shown in Figure 1b and details on construct design for Trx–TM3/4^Q220R^ and Trx–subunit C can be found in Figure 2a and Figure 3a, respectively.

We expressed the two fusion proteins in *Escherichia coli* BL21 (DE3), harvested and lysed the cells, clarified the lysate, and purified the His‐tagged Trx fusion proteins by IMAC (see Methods). As both fusion constructs harbor transmembrane proteins as fusion partners, the buffers contained Triton X‐100 to aid in solubilization. The elution fractions were concentrated and heat treated at varying temperatures and incubation times. In one series of experiments, we treated the samples at temperatures between 50 and 90°C for a fixed incubation time of 10 min. In another set of experiments, we heat treated the samples for 1–60 min at 70°C. In addition, we probed if the fusion proteins can be selectively purified from *E. coli* crude extract, similar to previous protocols.^19^, ^20^, ^21^ Crude purification was done by lysing cells expressing the two Trx fusion proteins and clarifying the cell lysate by centrifugation. The soluble fraction was then incubated at 50–90°C for 10 min.

For all procedures, we subjected the heat‐treated sample to SDS‐PAGE and quantitatively analyzed the band intensities (see Methods). Specifically, we assessed purity and recovery of band intensity of the respective Trx fusion proteins. Purity represents the fraction of intensity of the band of interest (e.g., Trx–TM3/4^Q220R^ or Trx–subunit C) over the total intensity of all bands in one sample (i.e., entire lane). Recovery denotes the fraction of intensity of the band of interest in heat‐treated samples over the intensity of the band of interest in the untreated sample (e.g., elution fraction), that is, the amount of protein that remains soluble after heat treatment.

For Trx–TM3/4^Q220R^ (Figure 2), sample purity in the untreated elution fraction after IMAC amounted to ~66%. Incubation at a rather low temperature of 50°C already markedly increased the purity of the fusion protein to ~91% (Figure 2b,c). Raising the temperature further in the heat treatment increased the purity of the fusion construct, maxing out at 70°C with remarkable ~98% purity. However, incubation at higher temperatures was also accompanied by a substantial reduction in recovery of the TM3/4 construct (Figure 2b,c). At 70°C, only ~65% of the initial Trx–TM3/4^Q220R^ band intensity remained. A further increase of temperature gradually reduced the recovery of the fusion construct to as low as ~21% at 90°C. The loss of Trx–TM3/4^Q220R^ is clearly visible in the lanes for the temperature range between 70 and 90°C (Figure 2b). Overall, our findings suggests that protein contaminants can be efficiently removed from elution fractions by denaturation and precipitation using the heat treatment protocol over the entire temperature range, with Trx–TM3/4^Q220R^ still remaining soluble. Nonetheless, elevated temperatures above 70°C reduce the solubility of Trx–TM3/4^Q220R^ markedly. Altering incubation times for the heat purification of Trx–TM3/4^Q220R^ at 70°C showed that a purity of ~95% can already be reached after just 1 min of incubation (Figure 2c,d). Accordingly, longer incubation periods did not greatly increase the purity of the elution fraction but did decrease the recovery of Trx–TM3/4^Q220R^ to ~60% after 60 min of incubation.

For the Trx–subunit C fusion (Figure 3), the untreated elution fraction showed a purity of ~52% for the monomeric Trx–subunit C band after IMAC (Figure 3b,c). Notably, an intense band at a molecular weight of >250 kDa was present as well (Figure 3b). A possible explanation for this high molecular weight band may be native oligomers or aggregates of Trx–subunit C, which did not migrate deeply into the gel, since subunit c is more prone to form aggregates when heterologously expressed.^26^ These oligomeric rings and aggregates of subunit c are even resistant to boiling in SDS, especially in presence of Na^+^ ions.^27^, ^28^ However, in comparison to subunit C without Trx‐tag,^27^, ^28^ Trx‐subunit C is largely present in a monomeric state (Figure 3b), suggesting that Trx prevents aggregation and aids in retaining subunit c as a soluble monomer. A strong increase in purity of Trx–subunit C as well as a loss of the band at >250 kDa occurred at temperatures above 70°C, peaking at 80°C with a purity of ~97% (Figure 3b,c). Thus, heating Trx‐subunit C in Triton X‐100‐containing elution buffer (see Methods) at >70°C is an effective approach to remove any aggregates preceding other studies, as they could, for example, remain to some extent even after boiling in SDS‐loading buffer (Figure 3b). The heat treatment, however, was again accompanied by a decrease in recovery (e.g., to ~41% recovery at 70°C). Remarkably, Trx–subunit C seems to be less heat stable than Trx–TM3/4^Q220R^, as the Trx–subunit C band showed only ~10% recovery at 80°C and only ~1% recovery at 90°C (Figure 3b,c). When varying incubation times at 70°C (Figure 3b,d), purity of Trx–subunit C could only be increased by treatment durations longer than 5 min, with purity of Trx–subunit C plateauing at ~90% and recovery dropping from ~51% after 5 min of treatment to ~20% after 60 min of treatment.

In addition, we investigated whether the heat treatment of the fusion constructs had an impact on their secondary structure by employing CD spectroscopy (Figure S1). For this purpose, the buffer of the respective Trx‐tagged construct was exchanged to a buffer with a composition suitable for CD analysis (see Methods). At 20°C, the CD spectra of both fusion proteins, Trx–TM3/4^Q220R^ and Trx–subunit C, showed a negative band between 208 and 222 nm, with a distinct peak at ~208 nm, typical for α‐helical proteins.^29^ While Trx is an α/β protein,^30^ TM3/4 and subunit C are both α‐helical peptide hairpins,^31^, ^32^ thus the fusion proteins are expected to mostly have a signature characteristic of an α‐helical structure. Upon temperature increase from 20 to 90°C, the ellipticity of both fusion proteins only slightly increased, as quantified for ellipticity values at 208 and 222 nm (Figure S1). Especially Trx–TM3/4^Q220R^ appeared to be resistant to thermal unfolding, while Trx–subunit C's structure appeared to be more affected at high temperatures (80–90°C), in agreement with our observation of reduced recovery at elevated temperatures for Trx–subunit C (Figure 3). Overall, our results from CD experiments indicate that Trx’ exceptional conformational stability over a wide temperature range^33^ is indeed shared with its transmembrane fusion proteins and secondary structure is preserved.

We further asked whether the Trx‐tagged transmembrane constructs can be selectively purified from *E. coli* crude extract, to circumvent affinity purification, as previously shown for soluble proteins such as growth factors and cytokines.^19^, ^20^, ^21^ To this end, we lysed *E. coli* cells expressing Trx–TM3/4^Q220R^ and Trx–subunit C and clarified the cell lysate by centrifugation. Samples of the cell pellet, cell lysate, and soluble fraction of the respective expression were analyzed by SDS‐PAGE (Figure S2). The soluble fraction was then subjected to heat treatment at different temperatures ranging from 50 to 90°C for 10 min. For Trx–TM3/4^Q220R^, purity only increased from ~18% of the untreated sample to around ~37–38% at 60 and 70°C, while the recovery dropped to ~82 and ~ 62% at 60 and 70°C, respectively (Figure S2). Higher temperatures yielded an even lower recovery, as low as ~2% at 90°C. We reason that this effect is caused by a co‐precipitation of Trx–TM3/4^Q220R^ with heat‐labile proteins that are still present in the soluble fraction, even to an extent where Trx–TM3/4^Q220R^ is more precipitated than some contaminants, which could explain the drop in purity of Trx–TM3/4^Q220R^ at 80°C and 90°C. Likewise, a selective purification of Trx–subunit C from the soluble fraction of an *E. coli* cell lysate could not be achieved (Figure S2), as heat treatment with increasing temperatures only reduced the purity and recovery of Trx–subunit C. We suspect that similar to Trx–TM3/4^Q220R^, co‐precipitation of Trx–subunit C with other lysate proteins decreases the recovery strongly.

## 3 CONCLUSIONS

We have demonstrated here that the purity of Trx‐tagged transmembrane constructs can be considerably improved, up to ~98%, by heat treatment after initial purification steps such as affinity chromatography. To maximize purity and recovery of Trx–TM3/4^Q220R^ from IMAC elution fractions, heat treatment could either be performed at lower temperatures for longer incubation periods (e.g., at 50°C for 10 min) or, complementary, at higher temperatures for shorter durations (e.g., at 70°C for 1 min). To increase purity of Trx–subunit C, optimal heat treatment conditions were found at 70°C for 5 min, to efficiently remove aggregates and other contaminants, while retaining a sufficient amount of the fusion protein. We observed that the Trx‐subunit C fusion protein (Trx: 11.8 kDa + subunit C: 8.7 kDa) was less thermostable than Trx–TM3/4^Q220R^ (Trx: 11.8 kDa + Q220R TM3/4: 5.1 kDa), suggesting that the heat treatment approach could be limited by the size of the fusion partner. It is possible that the thermostable properties conferred by Trx are attenuated when using a bigger interaction partner, though this remains to be explored. We have further demonstrated that the secondary structure integrity of the Trx fusion constructs is maintained at these temperatures. While selectively purifying Trx fusion constructs from whole‐cell crude extracts, as demonstrated previously,^19^, ^20^, ^21^ is conceptually straightforward, it may only be feasible for certain classes of well‐soluble proteins. Our results suggest that helical membrane protein constructs evade such a strategy. Therefore, selective purification of membrane proteins, as demonstrated here, can be achieved by heat treatment after purification by for example, affinity chromatography. We expect that our one‐step protocol for increasing the purity of Trx fusion constructs will be beneficial for many applications where high membrane protein purity is required, for example, in crystallization attempts for structural studies^9^ or in investigations of membrane proteins by single‐molecule techniques.^34^ We anticipate that the presented purification system might be also applicable to intrinsically disordered proteins or unstable non‐membrane proteins.

## 4 MATERIALS AND METHODS

Trx–TM3/4^Q220R^ was expressed from a pET32a expression vector (Merck) and the Trx–subunit C fusion construct was expressed from a pET24a expression vector (GenScript). Plasmids harboring the fusion constructs were transformed into *E. coli* One Shot BL21 Star (DE3) cells (Life Technologies). Expression was performed according to our previously described protocol.^22^ In brief, starter cultures were grown in Luria–Bertani (LB) medium, supplemented with 100 μg/ml ampicillin (for pET32a) or 50 μg/ml kanamycin (for pET24a), at 37°C overnight. These cultures were used to inoculate expression cultures in M9 minimal medium prepared from M9 minimal salt and containing 1 mM MgSO_4_, 0.1 mM CaCl_2_, 0.001% (wt/vol) thiamine, 0.001% (wt/vol) biotin, 3 g/L glucose and the respective antibiotic concentration as denoted above. Cell cultures were further cultivated at 37°C and induced at an OD of ~0.6–0.7 with a final concentration of 1 mM IPTG, followed by protein expression at 20°C overnight with subsequent centrifugation to harvest the cells. Pellets were washed with PBS and stored at −80°C or immediately used for protein purification.

Purification was initiated by thawing the pellets on ice with subsequent resuspension in lysis buffer (20 mM Tris–HCl, 150 mM NaCl, 0.1% (wt/vol) Triton X‐100, 10 mM β‐mercaptoethanol, 5 mM imidazole, pH 8.0) under addition of 0.1 μl/ml Benzonase (Merck). Cells were lysed with an Emulsiflex high‐pressure homogenizer (Avestin) or by sonication using a MS 73 sonication tip (Bandelin). The lysate was clarified by centrifugation and the soluble fraction was added to a Ni‐NTA resin (Qiagen) which was previously equilibrated with binding buffer (20 mM Tris–HCl, 150 mM NaCl, 0.1% (wt/vol) Triton X‐100, 10 mM β‐mercaptoethanol, 5 mM imidazole, pH 8.0). The lysate‐resin mixture was incubated under rotation for 90 min at room temperature and transferred into gravity flow columns to perform IMAC. The resin was washed stepwise with 20 mM Tris–HCl, 300 mM NaCl, 0.1% (wt/vol) Triton X‐100, 10 mM β‐mercaptoethanol, pH 8.0, supplemented with 5, 15, 25, and 50 mM imidazole. A final wash with binding buffer (containing 50 mM imidazole) was performed and His‐tagged Trx fusion proteins were eluted with elution buffer (20 mM Tris–HCl, 150 mM NaCl, 0.1% (wt/vol) Triton X‐100, 10 mM β‐mercaptoethanol, 400 mM imidazole, pH 8.0). Protein expression and purification was checked by SDS‐PAGE. PageRuler Plus Prestained Protein Ladder (Thermo Fisher) was used as a molecular weight marker for all SDS‐PAGE analyses. Elution fractions were stored at −80°C until further use.

Subsequently, elution fractions were thawed on ice and concentrated with a Vivaspin 6 centrifugal concentrator (5 kDa molecular weight cut‐off (MWCO), Sartorius) for 3–3.5 hr at 7,000*g* and 4°C; yielding a 3.4‐times concentrated elution fraction for Trx–TM3/4^Q220R^ (6 to 1.75 ml) and a five‐times concentrated elution fraction for Trx‐subunit C (6 to 1.2 ml), which were stored at −20°C if not used immediately. Afterwards, the concentrated elution fractions were aliquoted (each 40 μl) and heat‐treated for 10 min at 50–90°C (10°C steps, temperature series) or at 70°C for 1, 5, 10, 20, or 60 min (time series). Heat treated samples were then immediately centrifuged for 10 min at 20,817*g*, 4°C and the supernatants of each series were analyzed on a 12% SDS‐PA gel, after boiling (95°C, 10 min), loading equal sample amounts. To assess if heat treatment of Trx fusion constructs can be performed directly from the soluble fraction of a cell lysate, both fusion constructs were expressed and purified again as stated above (according to our previous protocol)^22^ until obtaining the soluble fraction of the cell lysates, taking additional samples from the cell pellet, lysate and soluble fraction. A heat treatment series was then performed with aliquots of the soluble fraction (each 1 ml) for 10 min at 50–90°C (10°C steps). Heat treated samples were centrifuged for 10 min at 20,817*g* and the supernatants were analyzed together with the pellet, lysate, and the soluble fraction on a 12% SDS‐PA gel, after boiling (95°C, 10 min), loading equal sample amounts. Ultimately, all gels were scanned with a resolution of 600 dpi using a CanoScan 9000F flatbed scanner (Canon) and saved as tagged image file format (TIF) files. Gel images were analyzed manually with the gel analyzer function of Fiji (ImageJ 1.52p).^35^ Before analysis, gel images were converted to 8‐bit grayscale images and a rolling ball background subtraction with a pixel size of 400 was performed to increase contrast.

To perform CD spectroscopy, the buffer of non‐concentrated or concentrated elution fractions of the fusion constructs was exchanged to CD buffer (20 mM Tris, 0.3% [wt/vol] SDS, pH 7.0) in several steps by employing His SpinTrap columns (GE Healthcare) and dialysis using Slide‐A‐Lyzer 10 K dialysis cassettes (10 kDa MWCO, Thermo Fisher). Protein concentration was estimated by SDS‐PAGE by comparing band intensities to a bovine serum albumin (BSA) standard row with known concentrations. Dialyzed samples were stored at −20°C until CD spectroscopy was performed. CD spectra were recorded employing a Chirascan‐plus spectrometer (Applied Photophysics) in the far‐UV range between a wavelength of 195–260 nm with a step size of 0.5 nm, a spectral bandwidth of 1 nm, a scanning speed of 30 nm per min, and a digital integration time of 1 s. The measurement temperature was raised from 20 to 90°C in 10°C increments. Samples were analyzed in a 1‐mm path length quartz glass cuvette, blanked against CD buffer (20 mM Tris, 0.3% [wt/vol] SDS, pH 7.0) and spectra were scanned five times and averaged.

## AUTHOR CONTRIBUTIONS

**Mathias Schenkel:** Conceptualization; data curation; formal analysis; investigation; methodology; validation; visualization; writing ‐ original draft; writing‐review & editing. **Antoine Treff:** Conceptualization; investigation; validation; writing‐review & editing. **Charles M. Deber:** Conceptualization; resources; validation; writing‐review & editing. **Georg Krainer:** Conceptualization; data curation; methodology; supervision; validation; writing‐original draft; writing‐review & editing. **Michael Schlierf:** Funding acquisition; project administration; resources; supervision; validation; writing‐review & editing.

## Supporting information

**Figure S1 CD spectra of Trx–TM3/4**^**Q220R**^**and Trx–subunit C at different temperatures. (A)** CD spectra of Trx–TM3/4^Q220R^ at 20–90°C. **(B)** Ellipticity at 208 nm (blue) and 222 nm (magenta) of Trx–TM3/4^Q220R^ measured at 20–90°C. **(C)** CD spectra of Trx–subunit C measured at 20–90°C. **(D)** Ellipticity at 208 nm (blue) and 222 nm (magenta) of Trx–subunit C at 20–90°C. Ellipticity values are averages of five spectra scans (panel A–D) and errors are standard deviations calculated from five spectra scans (panel B, D). The temperature range is color coded (panel A, C).
**Figure S2. Heat treatment of Trx–TM3/4**^**Q220R**^**and Trx–subunit C from *E. coli* crude lysate. (A)** 12% SDS‐PA gel with heat treatment series of Trx–TM3/4^Q220R^ purification samples from *E. coli*. Lanes show cell pellet (P), cell lysate (L), soluble lysate fraction (SF) after centrifugation and heat incubations for 10 min performed with the soluble lysate fraction at different temperatures. Molecular weight marker (M) and expected molecular weight of the Trx–TM3/4^Q220R^ band (~23.5 kDa, black arrow) are indicated. **(B)** Purity and recovery of the Trx‐TM3/4^Q220R^ band after heat treatment of the soluble lysate fraction at different temperatures for 10 min. Values are normalized with respect to the untreated sample. **(C)** 12% SDS‐PA gel with heat treatment series of Trx‐subunit C purification samples from *E. coli*. Lanes show cell pellet (P), cell lysate (L), soluble lysate fraction (SF) after centrifugation and heat incubations for 10 min performed with the soluble lysate fraction at different temperatures. Molecular weight marker (M) and expected molecular weight of the Trx–subunit C band (~25.8 kDa, black arrow) are indicated. **(D)** Purity and recovery of the Trx–subunit C band after heat treatment of the soluble lysate fraction at different temperatures for 10 min. Values are normalized with respect to the untreated sample.
