# Supplementary material for: Heat treatment of thioredoxin fusions increases the purity of α‐helical transmembrane protein constructs
Source: Protein Sci. 2021 Jul 6;30(9):1974–82. doi: 10.1002/pro.4150 (PMC8376418; doi:10.1002/pro.4150)
Supplement: Supplementary file 1 — Figure S1 CD spectra of Trx–TM3/4Q220Rand Trx–subunit C at different temperatures. (A) CD spectra of Trx–TM3/4Q220R at 20–90°C. (B) Ellipticity at 208 nm (blue) and 222 nm (magenta) of Trx–TM3/4Q220R measured at 20–90°C. (C) CD spectra of Trx–subunit C measured at 20–90°C. (D) Ellipticity at 208 nm (blue) and 222 nm (magenta) of Trx–subunit C at 20–90°C. Ellipticity values are averages of five spectra scans (panel A–D) and errors are standard deviations calculated from five spectra scans (panel B, D). The temperature range is color coded (panel A, C). Figure S2. Heat treatment of Trx–TM3/4Q220Rand Trx–subunit C from E. coli crude lysate. (A) 12% SDS‐PA gel with heat treatment series of Trx–TM3/4Q220R purification samples from E. coli. Lanes show cell pellet (P), cell lysate (L), soluble lysate fraction (SF) after centrifugation and heat incubations for 10 min performed with the soluble lysate fraction at different temperatures. Molecular weight marker (M) and expected molecular weight of the Trx–TM3/4Q220R band (~23.5 kDa, black arrow) are indicated. (B) Purity and recovery of the Trx‐TM3/4Q220R band after heat treatment of the soluble lysate fraction at different temperatures for 10 min. Values are normalized with respect to the untreated sample. (C) 12% SDS‐PA gel with heat treatment series of Trx‐subunit C purification samples from E. coli. Lanes show cell pellet (P), cell lysate (L), soluble lysate fraction (SF) after centrifugation and heat incubations for 10 min performed with the soluble lysate fraction at different temperatures. Molecular weight marker (M) and expected molecular weight of the Trx–subunit C band (~25.8 kDa, black arrow) are indicated. (D) Purity and recovery of the Trx–subunit C band after heat treatment of the soluble lysate fraction at different temperatures for 10 min. Values are normalized with respect to the untreated sample. [file PRO-30-1974-s001.pdf]

Supporting information for

Heat treatment of thioredoxin fusions increases the purity of α-helical transmembrane protein constructs

Mathias Schenkel,[1] Antoine Treff,[1] Charles M. Deber,[2] Georg Krainer,[1,3,*] Michael Schlierf,[1,*]

[1] B CUBE – Center for Molecular Bioengineering, TU Dresden, Tatzberg 41, 01307 Dresden, Germany

[2] Division of Molecular Medicine, Research Institute, Hospital for Sick Children, 686 Bay Street, Toronto, ON M5G 0A4, Canada

[3] Centre for Misfolding Diseases, Yusuf Hamied Department of Chemistry, University of Cambridge, Lensfield Road, CB2 1EW Cambridge, UK

** To whom correspondence should be addressed: michael.schlierf@tu-dresden.de (Mi.S.) and gk422@cam.ac.uk (G.K.)*

SUPPORTING FIGURES

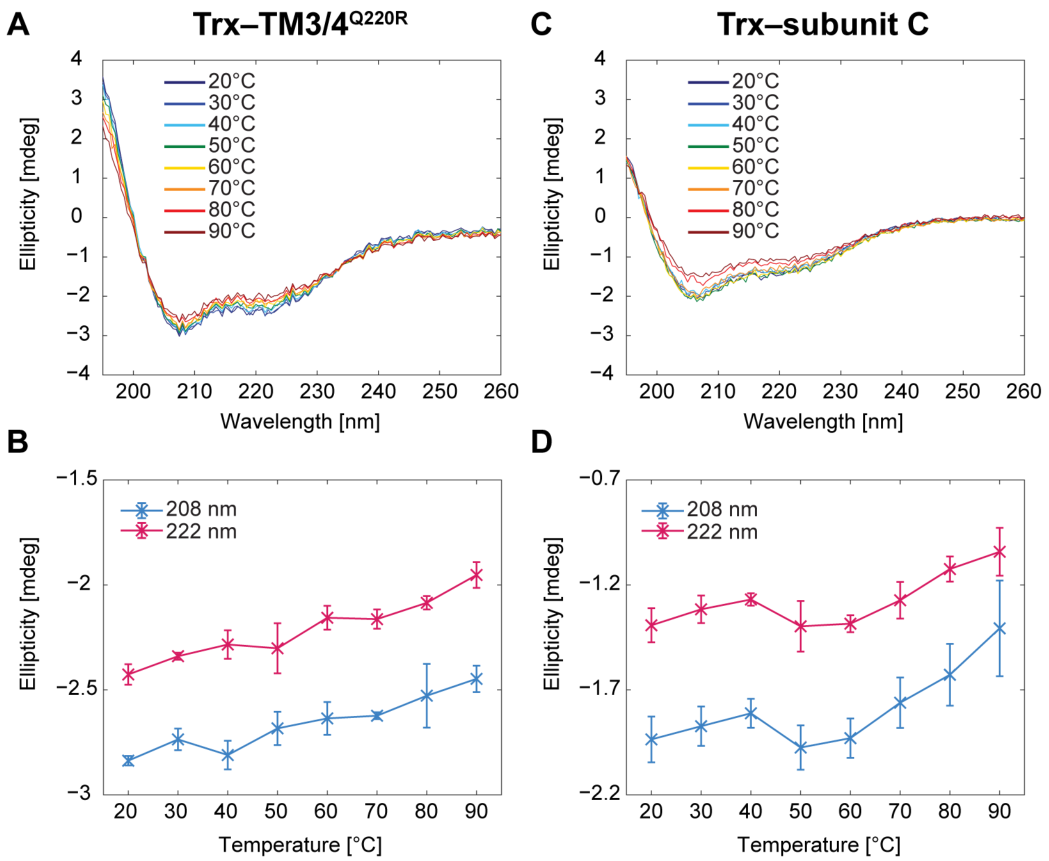

Figure S1. CD spectra of Trx–TM3/4^{Q220R} and Trx–subunit C at different temperatures. (A) CD spectra of Trx–TM3/4^{Q220R} at 20–90°C. **(B)** Ellipticity at 208 nm (blue) and 222 nm (magenta) of Trx–TM3/4^{Q220R} measured at 20–90°C. **(C)** CD spectra of Trx–subunit C measured at 20–90°C. **(D)** Ellipticity at 208 nm (blue) and 222 nm (magenta) of Trx–subunit C at 20–90°C. Ellipticity values are averages of five spectra scans (panel A–D) and errors are standard deviations calculated from five spectra scans (panel B, D). The temperature range is color coded (panel A, C).

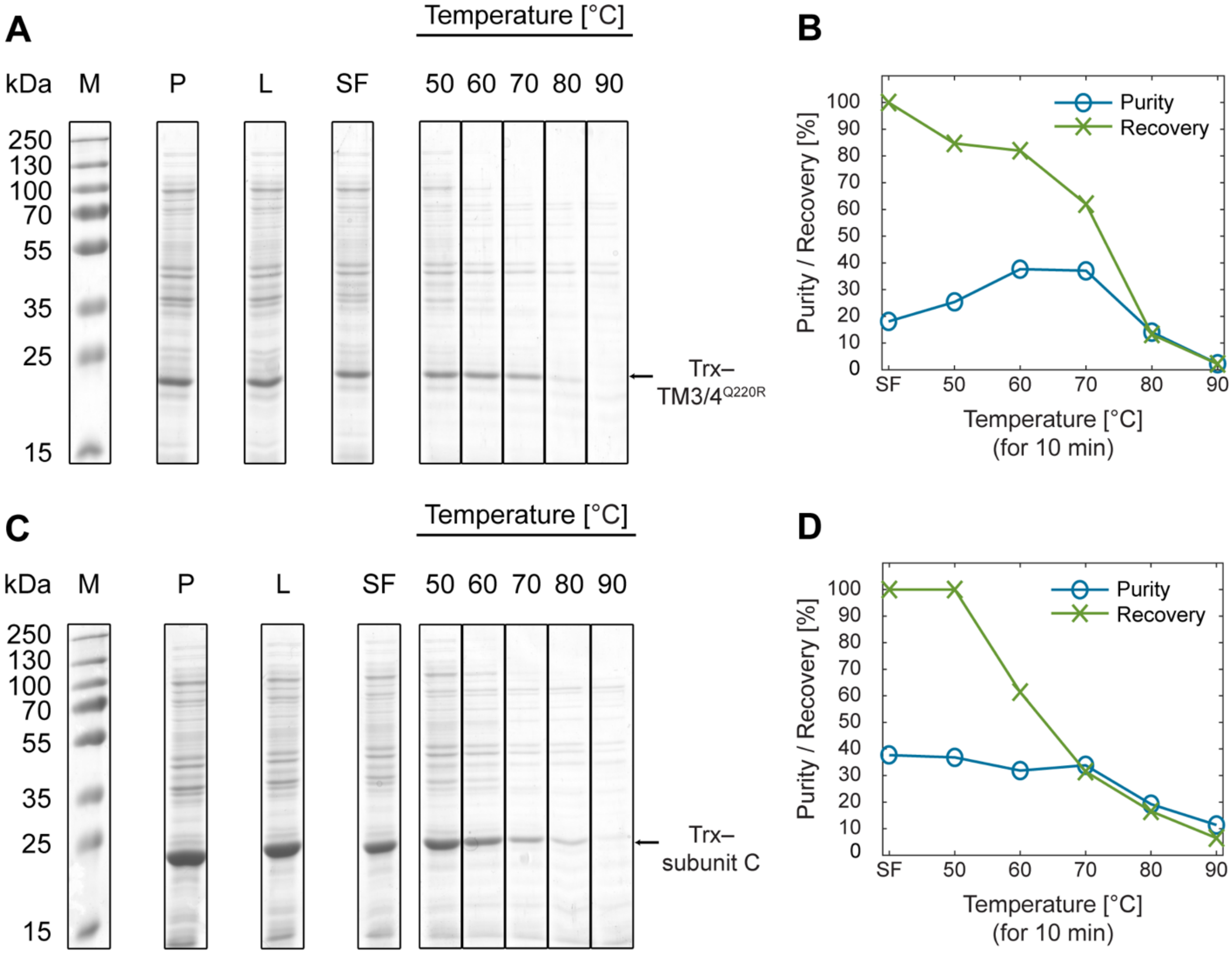

Figure S2. Heat treatment of Trx–TM3/4^{Q220R} and Trx–subunit C from *E. coli* crude lysate. (A) 12% SDS-PA gel with heat treatment series of Trx–TM3/4^{Q220R} purification samples from *E. coli*. Lanes show cell pellet (P), cell lysate (L), soluble lysate fraction (SF) after centrifugation and heat incubations for 10 min performed with the soluble lysate fraction at different temperatures. Molecular weight marker (M) and expected molecular weight of the Trx–TM3/4^{Q220R} band (~23.5 kDa, black arrow) are indicated. **(B)** Purity and recovery of the Trx-TM3/4^{Q220R} band after heat treatment of the soluble lysate fraction at different temperatures for 10 min. Values are normalized with respect to the untreated sample. **(C)** 12% SDS-PA gel with heat treatment series of Trx-subunit C purification samples from *E. coli*. Lanes show cell pellet (P), cell lysate (L), soluble lysate fraction (SF) after centrifugation and heat incubations for 10 min performed with the soluble lysate fraction at different temperatures. Molecular weight marker (M) and expected molecular weight of the Trx–subunit C band (~25.8 kDa, black arrow) are indicated. **(D)** Purity and recovery of the Trx–subunit C band after heat treatment of the soluble lysate fraction at different temperatures for 10 min. Values are normalized with respect to the untreated sample.
